# Predicting drug-disease associations by using similarity constrained matrix factorization

**DOI:** 10.1186/s12859-018-2220-4

**Published:** 2018-06-19

**Authors:** Wen Zhang, Xiang Yue, Weiran Lin, Wenjian Wu, Ruoqi Liu, Feng Huang, Feng Liu

**Affiliations:** 1 School of Computer Science, Wuhan University, Wuhan, 430072 China; 2 School of Electronic Information, Wuhan University, Wuhan, 430072 China

**Keywords:** Drug-disease associations, Similarity constrained matrix factorization

## Abstract

**Background:**

Drug-disease associations provide important information for the drug discovery. Wet experiments that identify drug-disease associations are time-consuming and expensive. However, many drug-disease associations are still unobserved or unknown. The development of computational methods for predicting unobserved drug-disease associations is an important and urgent task.

**Results:**

In this paper, we proposed a **s**imilarity **c**onstrained **m**atrix **f**actorization method for the **d**rug-**d**isease association prediction (SCMFDD), which makes use of known drug-disease associations, drug features and disease semantic information. SCMFDD projects the drug-disease association relationship into two low-rank spaces, which uncover latent features for drugs and diseases, and then introduces drug feature-based similarities and disease semantic similarity as constraints for drugs and diseases in low-rank spaces. Different from the classic matrix factorization technique, SCMFDD takes the biological context of the problem into account. In computational experiments, the proposed method can produce high-accuracy performances on benchmark datasets, and outperform existing state-of-the-art prediction methods when evaluated by five-fold cross validation and independent testing.

**Conclusion:**

We developed a user-friendly web server by using known associations collected from the CTD database, available at http://www.bioinfotech.cn/SCMFDD/. The case studies show that the server can find out novel associations, which are not included in the CTD database.

## Background

A drug is a chemical that treats, cures, prevents, or diagnoses diseases. The drug design has three stages: discovery stage, preclinical stage and clinical development stage [1], and the development of a new drug take 15 years [2] and cost 800 million dollars [3].

The drug-disease associations refer to the events that drugs exert effects on diseases, which can be classified into two types: drug indications and drug side-effects. Some drugs could have a therapeutic role in a disease, e.g. a drug treats leukemia & lymphoma; other drugs could play a role in the etiology of a disease, e.g. exposure to a drug causes lung cancer [4]. Drug-disease associations reveal the close relations between drugs and diseases, and have gained great attention. Computational methods can screen possible drug-disease associations, and complement or guide laborious and costly wet experiments.

**Fig. 1:**
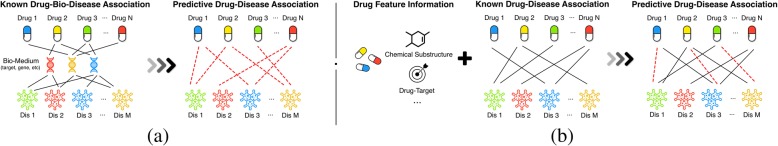
Two types of drug-disease association prediction methods. **a** Infer drug-disease associations without known associations; **b** Infer unobserved drug-disease associations based on known associations

In recent years, a great number of computational methods have been proposed to predict drug-disease associations. As shown in Fig. 1, existing methods are roughly classified as two types. One type of methods makes use of biological elements shared by drugs and diseases to predict drug-disease associations. Eichborn J et al. [5] studied drug-disease relations based on drug side effects. Wang et al. [6] and Wiegers et al. [7] considered drug-gene-disease relations. Yu et al. [8] used common protein complexes related to drugs and diseases. These methods have to use elements shared by drugs and diseases, but many drugs and diseases do not share any elements, and these methods fail to work in this case. The other type of methods predicts novel drug-disease associations by using known drug-disease associations, drug features and disease features. Gottlieb et al. [9] constructed a universal predictor named PREDICT for drug repositioning to express drug-disease associations in a large-scale manner that integrated molecular structure, molecular activity and disease semantic data. Yang et al. [10] built Naive Bayes models to predict indications for diseases based on their side effects. Wang et al. [11] proposed the method “PreDR” that trained a support vector machine (SVM) model based on drug structures, drug target proteins, and drug side effects. Huang et al. [12] combined three different networks of drugs, genomic and disease phenotypes to build a heterogeneous network to predict drug-disease associations. Oh et al. [13] proposed scoring methods to obtain quantified scores as features between drugs and diseases, and built classifiers based on the extracted features to predict novel drug-disease associations. Wang et al. [14] proposed a three-layer heterogeneous network model (TL-HGBI), and applied the approach on drug repositioning by using existing omics data of diseases, drugs and drug targets. Martínez et al. [15] built a network of interconnected drugs, proteins and diseases to identify their relations. Wang et al. [16] adopted recommendation systems to predict drug-disease relations. Moghadam et al. [17] combined drug features and disease features by using kernel fusion, and then built SVM-based prediction model. Liang et al. [18] proposed a Laplacian regularized sparse subspace learning method (LRSSL), which integrated drug chemical information, drug target domain information and target annotation information.

A great number of drug-disease associations have been identified and stored in databases. However, many associations remain unobserved and need to be discovered. In this paper, we proposed a **s**imilarity **c**onstrained **m**atrix **f**actorization method for the **d**rug-**d**isease association prediction (SCMFDD), which makes use of known drug-disease associations, drug features and disease semantic information. SCMFDD projects the drug-disease association relationship into two low-rank spaces, which uncover latent features for drugs and diseases, and then introduces drug feature-based similarity and disease semantic similarity as constraints for drugs and diseases in low-rank spaces. Different from the classic matrix factorization technique, SCMFDD can take the biological context of the problem into account. Computational experiments show that SCMFDD can produce high-accuracy performances on benchmark datasets and outperform existing state-of-the-art prediction methods, i.e. PREDICT, TL-HGBI and LRSSL when evaluated by five-fold cross validation and independent testing on the same datasets. Moreover, a web server is constructed on known associations collected from the CTD database [4], and case studies show that the web server can help to find out novel associations.

The main contributions of this paper include: 1) we proposed a novel matrix factorization approach (SCMFDD), which is different from the traditional matrix factorization methods. SCMFDD incorporates drug features and disease semantic information into the matrix factorization frame; 2) an efficient optimization algorithm is developed to obtain the solution of SCMFDD; 3) we developed a user-friendly web server to facilitate the drug-disease association prediction, available at http://www.bioinfotech.cn/SCMFDD/.

## Methods

### Datasets

CTD database [4] is a publicly available database that intends to advance understanding about how environmental exposures affect human health. CTD database provides curated and inferred chemical-disease associations. The curated associations are real associations extracted from literature. Several databases describe features for drugs and diseases. PubChem Compound database [19] provides drug substructures. DrugBank database [20] is a comprehensive resource for drug targets, drug enzymes and drug-drug interactions. KEGG DRUG database [21] provides pathway information for approved drugs in Japan, USA and Europe. U.S. National Library of Medicine stores disease MeSH descriptors, which reflect the hierarchy of diseases.

We downloaded real drug-disease associations from CTD database, and collected features for drugs and diseases to compile our datasets. In order to avoid sparsity of drug-disease associations, we selected drugs that are associated with more than 10 diseases, and also selected diseases that are associated with more than 10 drugs. Moreover, we collected drug features: substructures, targets, enzymes, pathways and drug-drug interactions as well as disease MeSH descriptors. Thus, we compiled a dataset named “SCMFDD-S”, which contains 18,416 associations between 269 drugs and 598 diseases. Further, we selected drugs associated with at least one disease as well as diseases associated with at least one drug, and collected drug substructures and disease MeSH descriptors. Thus, we compiled a larger dataset named “SCMFDD-L”, which contains 49,217 associations between 1323 drugs and 2834 diseases. Table 1 summarizes the datasets “SCMFDD-S” and “SCMFDD-L”.

**Table 1:** The summary of SCMFDD-S dataset and SCMFDD-L dataset

Dataset	Drugs	Diseases	Associations	Richness	Drug features				
					Substructure	Target	Enzyme	Pathway	Drug Interactions
SCMFDD-S	269	598	18,416	11.4%	881	623	247	465	2086
SCMFDD-L	1323	2834	49,217	1.31%	881	N.A.	N.A.	N.A.	N.A.

Numbers for drug features represent the numbers of descriptors. For example, the PubChem Compound defines 881 types of substructure descriptors for compound substructures, and a drug has some substructures and is thus described by a subset of substructure descriptors. Richness is the ratio of association number vs drug-disease pair number. N.A. indicates that the information is not available

Several benchmark datasets were used in the drug-disease association prediction. Gottlieb et al. [9] compiled a dataset with 1933 associations between 593 drugs in DrugBank and 313 diseases in OMIM, and used it for the method “PREDICT”. This dataset contains five types of drug-drug similarities and two types of disease-disease similarities. Three drug-drug similarities are calculated based on drug-related genes, by using Smith-Waterman sequence alignment score [22], all-pairs shortest paths algorithm [23] and semantic similarity scores [24] respectively; other two drug-drug similarities are drug structure-based Tanimoto similarity and drug side effect-based Jaccard similarity. Two disease-disease similarity measures are semantic similarity and genetic similarity. Wang et al. [14] compiled a dataset with 1461 interactions between 1409 drugs in DrugBank database and 5080 diseases in OMIM database, and used it for the method “TL-HGBI”. The dataset also contains the drug-drug structure similarity and disease semantic similarity. Liang et al. [18] obtained 3051 associations between 763 drugs and 681 diseases from the study [25], and collected drug substructures, protein domains of target proteins, gene ontology terms of target proteins to calculate three types of drug-drug similarities as well as the disease-disease semantic similarity. The dataset was used for the method “LRSSL”. We name these datasets as “PREDICT dataset”, “TL-HGBI dataset” and “LRSSL datasets”.

Therefore, we adopt SCMFDD-S dataset, SCMFDD-L dataset, PREDICT dataset, TL-HGBI dataset and LRSSL datasets as benchmark datasets.

### Similarity constrained matrix factorization method

**Fig. 2:**
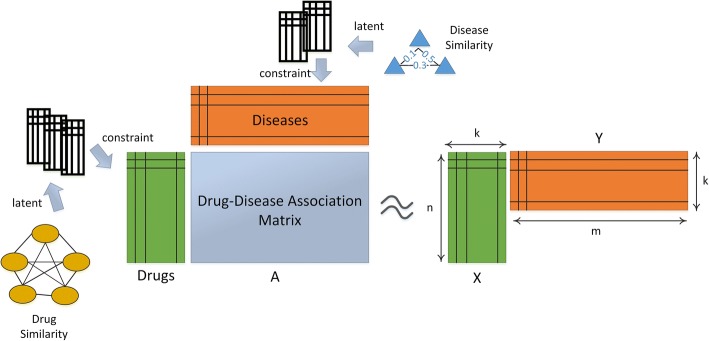
The basic idea of similarity constrained matrix factorization

The aim of this study is to predict unobserved drug-disease associations by using drug features, disease semantic information and known associations. Figure 2 illustrates the basic idea of the similarity constrained matrix factorization method for the drug-disease association prediction (SCMFDD).

#### Drug-drug similarities

Actually, a feature is a set of descriptors. A drug has a subset of descriptors, and thus is represented as a bit vector, whose dimensions indicate the presence or absence of corresponding descriptors with the value 1 or 0. Let *P* and *Q* denote feature vectors of two drugs, we can calculate the Jaccard similarity between two drugs by using,

$$ \mathrm{J}\left(\mathrm{P},\mathrm{Q}\right)=\frac{\mid \mathrm{P}\cap \mathrm{Q}\mid }{\mid \mathrm{P}\cup \mathrm{Q}\mid } $$

where P ∩ Q∣ is the number of bits where *P* and *Q* both have the value 1, and P ∪ Q∣ is the number of bits where either *P* and *Q* has the value 1.

When we have different features of a drug, i.e. substructures, targets, enzymes, pathways and drug-drug interactions, we can represent them as feature vectors in different feature spaces, and calculate different types of drug-drug similarities.

#### Disease-disease semantic similarity

MeSH is the National Library of Medicine’s controlled vocabulary thesaurus, and MeSH provides hierarchical descriptors for diseases. As described in [26–28], we can calculate disease-disease semantic similarity by using MeSH information.

For each disease, a directed acyclic graph (*DAG*) is constructed based on hierarchical descriptors, in which nodes represent disease MeSH descriptors (or disease terms) and the edges represent the relationship between the current node and its ancestors. For the disease *A*, the *DAG* is denoted as *DAG*(*A*) = (*N*(*A*), *E*(*E*)), where *N*(*A*) is the set of all ancestors of *A* (including itself) and *E*(*A*) is the set of their corresponding links.

We define the contribution of a node *d d* in *DAG*(*A*) to the semantic value of disease *A*:

$$ {C}_A(d)=\left\{\begin{array}{c}1\kern16.75em if\ d=A\\ {}\mathit{\max}\left\{\Delta  \ast {C}_A\left({d}^{\prime}\right)|{d}^{\prime}\in children\ of\ \left.d\right\}\kern0.5em if\ d\ne A\right.\end{array}\right. $$

where *∆* is the semantic contribution factor, and we set *∆* = 0.5 in the study.

The semantic value of disease *A* is defined as,

$$ DV(A)=\sum \limits_{d\in N(A)}{C}_A(d) $$

The semantic similarity between two diseases *A* and *B* is calculated by,

$$ {S}_{A,B}=\frac{\sum_{d\in N\left(\mathrm{A}\right)\cap N(B)}\left({C}_A(d)+{C}_B(d)\right)}{DV(A)+ DV(B)} $$

#### Objective Function

**Fig. 3:**
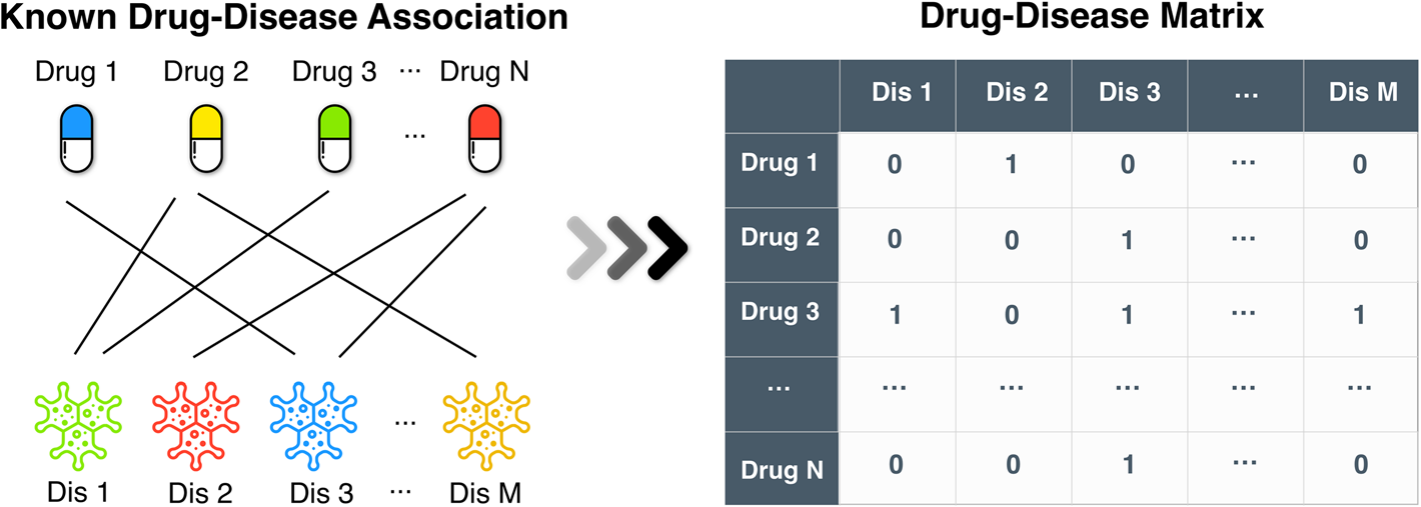
The bipartite network and the association network

The observed drug-disease associations can be formulated as a bipartite network, and represented by a binary matrix *A* ∈ *R*^*n* × *m*^, where *n* is the number of drugs and *m* is the number of diseases. *a*_*ij*_ is the (*i*, *j*)th entry of *A*. If the vertex (drug) *d*_*i*_ and the vertex (disease) *dis*_*j*_ are connected, *a*_*ij*_ = 1; otherwise *a*_*ij*_ = 0. The bipartite network and the association matrix are demonstrated in Fig. 3.

SCMFDD factorizes the drug-disease association matrix *A* into two low-rank feature matrices *X* ∈ *R*^*n* × *k*^ and *Y* ∈ *R*^*m* × *k*^, where *k* is the dimension of drug feature and disease feature in the low-rank spaces. The drug-disease association can be approximated by inner product between the drug feature vector and the disease feature vector: ${a}_{ij}\approx {\boldsymbol{x}}_i{\boldsymbol{y}}_j^T$, where ***x***_*i*_ is the *i*th row of *X*, and ***y***_*j*_ is the *j*th row of *Y*.The objective function is defined as:

1 $$ \mathit{\min}\frac{1}{2}\sum \limits_{ij}{\left({a}_{ij}-{\boldsymbol{x}}_i{\boldsymbol{y}}_j^T\right)}^2 $$

Then, to avoid overfitting problem, *L*_2_ regularization terms of ***x***_*i*_ and ***y***_*j*_ are added to the objective function (1),

2 $$ \mathit{\min}\frac{1}{2}\sum \limits_{ij}{\left({a}_{ij}-{\boldsymbol{x}}_i{\boldsymbol{y}}_j^T\right)}^2+\frac{\mu }{2}\sum \limits_i{\left\Vert {\boldsymbol{x}}_i\right\Vert}^2+\frac{\mu }{2}\sum \limits_j{\left\Vert {\boldsymbol{y}}_j\right\Vert}^2 $$

where *μ* is the regularization parameter for ***x***_*i*_ and ***y***_*j*_.

Recent studies on manifold learning theory [29, 30], spectral graph theory [31, 32] and their applications [33–38] show that the geometric and topological structure of data points may be maintained when they are mapped from high dimensional space into low dimensional space. Considering that the similarity matrix *w*^*d*^ and *w*^*s*^ not only can be defined to represent statistical correlation but also can be regarded as geometric properties of the data points, we introduce the similarity constraint terms *R*_*X*_ and *R*_*Y*_:

3 $$ {R}_X=\frac{1}{2}\sum \limits_{ij}{\left\Vert {\boldsymbol{x}}_i-{\boldsymbol{x}}_j\right\Vert}^2{w}_{ij}^d $$

4 $$ {R}_Y=\frac{1}{2}\sum \limits_{ij}{\left\Vert {\boldsymbol{y}}_i-{\boldsymbol{y}}_j\right\Vert}^2{w}_{ij}^s $$

where ${w}_{ij}^d$ denotes the similarity between the drug *d*_*i*_ and the drug *d*_*j*_, which is calculated in the drug feature space; ${w}_{ij}^s$ denotes the similarity between the disease *dis*_*i*_ and the disease *dis*_*j*_, which is calculated in the disease feature space. It is generally believed that the similarity between two data points is higher if the distance of them is smaller. Therefore, *R*_*X*_(or *R*_*Y*_) incurs a heavy penalty if drug *d*_*i*_ and the drug *d*_*j*_(disease *dis*_*i*_ and the disease *dis*_*j*_) are close in the drug feature space (or disease feature space) and thus minimizing it further incurs that drug *d*_*i*_ and the drug *d*_*j*_(or disease *dis*_*i*_ and the disease *dis*_*j*_) are mapped closely in low-rank spaces. Hence, we could maintain effectively the topological structure of drug data points and disease data points by minimizing *R*_*X*_ and *R*_*Y*_.

By combining *R*_*X*_ and *R*_*Y*_ with the original objective function (2), we propose the objective function of SCMFDD,

5 $$ \underset{X,Y}{\min }L=\frac{1}{2}\sum \limits_{ij}{\left({a}_{ij}-{\boldsymbol{x}}_i{\boldsymbol{y}}_j^T\right)}^2+\frac{\mu }{2}\sum \limits_i{\left\Vert {\boldsymbol{x}}_i\right\Vert}^2+\frac{\mu }{2}\sum \limits_j{\left\Vert {\boldsymbol{y}}_j\right\Vert}^2+\frac{\uplambda}{2}\sum \limits_{ij}{\left\Vert {\boldsymbol{x}}_i-{\boldsymbol{x}}_j\right\Vert}^2{w}_{ij}^d+\frac{\uplambda}{2}\sum \limits_{ij}{\left\Vert {\boldsymbol{y}}_i-{\boldsymbol{y}}_j\right\Vert}^2{w}_{ij}^s $$

where λ is the hyper parameter controlling the smoothness of the similarity consistency.

#### Optimization algorithm

Here, we develop an efficient optimization algorithm to solve the objective function in (5). First, we calculate the partial derivatives of *L* with respect to ***x***_*i*_ and ***y***_*j*_,

6 $$ {\nabla}_{{\boldsymbol{x}}_i}L=\sum \limits_j\left({\boldsymbol{x}}_i{\boldsymbol{y}}_j^T-{a}_{ij}\right){y}_j+{\mu \boldsymbol{x}}_i+\uplambda \left(\sum \limits_j\left({\boldsymbol{x}}_i-{\boldsymbol{x}}_j\right){w}_{ij}^d-\sum \limits_j\left({\boldsymbol{x}}_j-{\boldsymbol{x}}_i\right){w}_{ji}^d\right)={\boldsymbol{x}}_i\left({Y}^TY+\mu I+\uplambda \left(\sum \limits_j{w}_{ij}^d+\sum \limits_j{w}_{ji}^d\right)I\right)-A\left(i,:\right)Y-\uplambda \sum \limits_j\left({w}_{ij}^d+{w}_{ji}^d\right){\boldsymbol{x}}_j $$

7 $$ {\nabla}_{{\boldsymbol{y}}_j}L=\sum \limits_i\left({\boldsymbol{y}}_j{\boldsymbol{x}}_i^T-{a}_{ij}\right){x}_i+\mu {\boldsymbol{y}}_j+\uplambda \left(\sum \limits_i\left({\boldsymbol{y}}_j-{\boldsymbol{y}}_i\right){w}_{ji}^s-\sum \limits_i\left({\boldsymbol{y}}_i-{\boldsymbol{y}}_j\right){w}_{ij}^s\right)={\boldsymbol{y}}_j\left({X}^TX+\mu I+\uplambda \left(\sum \limits_i{w}_{ij}^s+\sum \limits_i{w}_{ji}^s\right)I\right)-A{\left(:,j\right)}^TX-\uplambda \sum \limits_i\left({w}_{ij}^s+{w}_{ji}^s\right){\boldsymbol{y}}_i $$

*A*(*i*, :) represents the *i*th row of *A* and *A*(:, *j*) represents the *j*th column of *A*.

Then, we can calculate the second derivatives of *L* with respect to ***x***_*i*_ and ***y***_*j*_:

8 $$ {\nabla}_{{\boldsymbol{x}}_i}^2L={Y}^TY+\mu I+\uplambda \left(\sum \limits_j{w}_{ij}^d+\sum \limits_j{w}_{ji}^d\right)I $$

9 $$ {\nabla}_{{\boldsymbol{y}}_j}^2L={X}^TX+\mu I+\uplambda \left(\sum \limits_i{w}_{ij}^s+\sum \limits_i{w}_{ji}^s\right)I $$

Utilizing Newton’s method, we have:

10 $$ {\boldsymbol{x}}_i\leftarrow {\boldsymbol{x}}_i-{\nabla}_{{\boldsymbol{x}}_i}L{\left({\nabla}_{{\boldsymbol{x}}_i}^2L\right)}^{-1} $$

11 $$ {\boldsymbol{y}}_j\leftarrow {\boldsymbol{y}}_j-{\nabla}_{{\boldsymbol{y}}_j}L{\left({\nabla}_{{\boldsymbol{y}}_j}^2L\right)}^{-1} $$

Thus, we can obtain the updating rules:

12 $$ {\boldsymbol{x}}_i=\left(A\left(i,:\right)Y+\uplambda \sum \limits_j\left({w}_{ij}^d+{w}_{ji}^d\right){\boldsymbol{x}}_j\right){\left({Y}^TY+\mu I+\uplambda \left(\sum \limits_j{w}_{ij}^d+\sum \limits_j{w}_{ji}^d\right)I\right)}^{-1} $$

13 $$ {\boldsymbol{y}}_j=\left(A{\left(:,j\right)}^TX+\uplambda \sum \limits_i\left({w}_{ij}^s+{w}_{ji}^s\right){\boldsymbol{y}}_i\right){\left({X}^TX+\mu I+\uplambda \left(\sum \limits_i{w}_{ij}^s+\sum \limits_i{w}_{ji}^s\right)I\right)}^{-1} $$

We alternatively update ***x***_*i*_ and ***y***_*j*_ with Eq. (12) and Eq. (13) until convergence. The prediction matrix is given by

14 $$ {A}_{predict}=X{Y}^T $$

The score of (*A*_*predict*_)_*ij*_ represents the probability that the drug *d*_*i*_ and the disease *dis*_*j*_ has the association. The optimization algorithm is summarized in Algorithm 1.

**Table:** 

**Algorithm 1** Algorithm to solve objective function (5)	
**Input:** known drug-disease association matrix, *A* ∈ *R*^*n* × *m*^; drug similarity matrix, *W*^*d*^ ∈ *R*^*n* × *n*^; disease similarity matrix, *W*^*s*^ ∈ *R*^*m* × *m*^; dimension of the low-rank feature space, *k* < min(*m*, *n*); regularization parameter, *μ* > 0, *λ* > 0; **Output:** the prediction matrix *A*_*predict*_ 1 Initialize *X* ∈ *R*^*n* × *k*^, *Y* ∈ *R*^*m* × *k*^ as two random matrices; 2 **Repeat** 3 **Update** *X*: 4 **for** each *i*(1 ≤ *i* ≤ *n*) **do** 5 update *x*_*i*_ by Eq. (12); 6 **end** 7 **Update** *Y*: 8 **for** each *j*(1 ≤ *j* ≤ *m*) **do** 9 update *y*_*j*_ by Eq. (13); 10 **end** 11 **Until** Converges; 12 Calculate the prediction matrix *A*_*predict*_ by Eq. (14); 13 Output *A*_*predict*_;	

## Results and discussion

### Evaluation metrics

In our experiments, we adopted five-fold cross validation (5-CV) to test performances of prediction models. To implement five-fold cross validation, we randomly split all known drug-disease associations into five equal-sized subsets. In each fold, we combined four subsets as the training set, and used the other subset as the testing set. We constructed the prediction model based on known associations in the training set, and predicted associations in the testing set. Training and testing were repeated five times, and the average of performances was adopted.

AUC and AUPR are popular metrics for evaluating prediction models. Since drug-disease pairs without associations are much more than known drug-disease associations, we adopted AUPR as the primary metric, which takes into recall and precision. We also considered several binary classification metrics, i.e. sensitivity (SN, also known as recall), specificity (SP), accuracy (ACC) and F-measure (F).

### Performances of SCMFDD

First of all, we discussed the influence of parameters on SCMFDD models by using SCMFDD-S dataset. SCMFDD has three parameters, i.e. the number of latent variables *k*, the regularization parameter *μ* and the regularization parameter λ. *k* is the dimension of drugs and diseases in low-rank spaces, and *k* is less than row number and column number of the association matrix, and *k* < *k*_0_ = min(*m*, *n*). For simplicity, we set *k* as the percentage of *k*_0_.

SCMFDD builds prediction model constrained by drug-drug similarity and disease-disease semantic similarity. We have several drug features in SCMFDD-S dataset, and can calculate several types of drug-drug similarities. Here, we used the drug interaction-based similarity and the disease semantic similarity to build SCMFDD models for analysis. We considered all combinations of following values λ ∈ {2^−3^, 2^−2^, 2^−1^, 2^0^, 2^1^, 2^2^, 2^3^}, *μ* ∈ {2^−3^, 2^−2^, 2^−1^, 2^0^, 2^1^, 2^2^, 2^3^} and *k* ∈ {5%, 10%, 15 % …, 50%} to build SCMFDD models, and implemented five-fold cross validation to evaluate models. The experiments for all parameter combinations cost about 12 h on a PC with Intel i7 7700 K CPU and 16GB RAM.

**Fig. 4:**
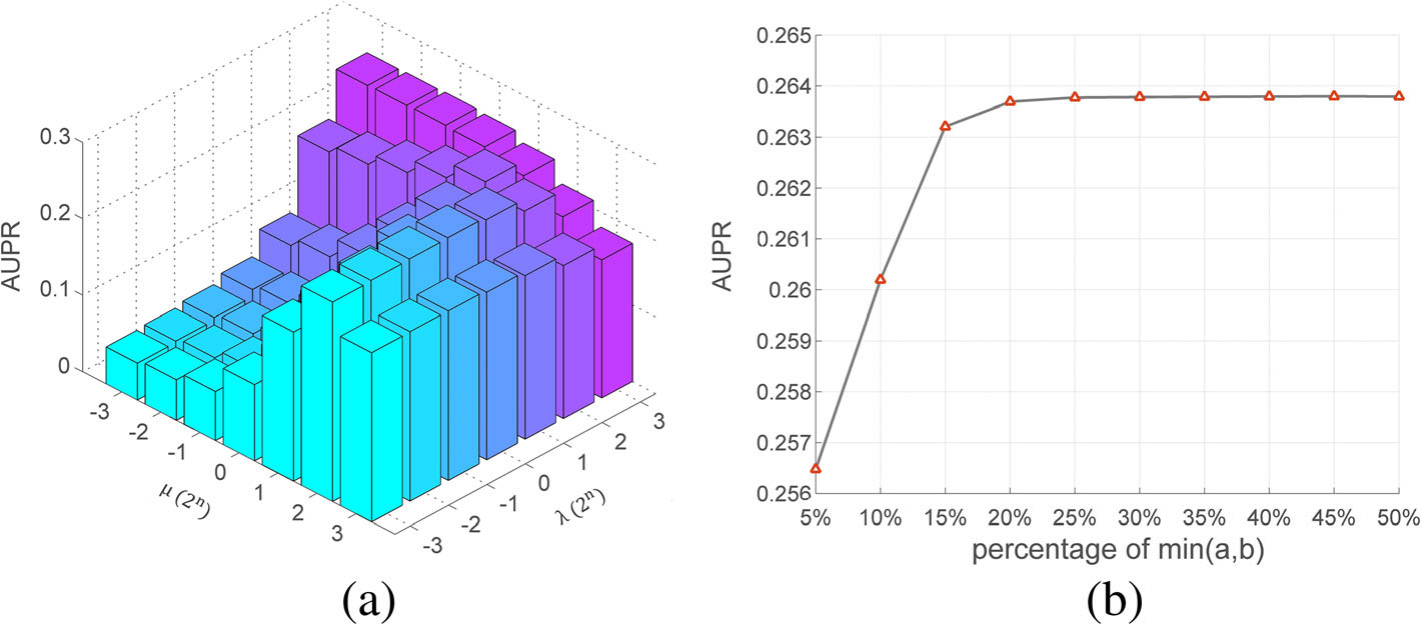
The influence of parameters on SCMFDD models. **a** the influnce of μ and λ **b** the influence of k

In computational experiments, SCMFDD produced the best AUPR score when *k* = 45 % ,  *μ* = 2^0^ and λ = 2^2^. Then, we fixed the latent variable number *k* = 45%, and evaluated the influence of parameters *μ* and λ, and results are shown in Fig. 4a. Clearly, *μ* and λ have great impact on the model. When *μ* is a small value, greater λ could lead to better performances; when *μ* is a great value, greater λ contributes to poorer performances. Further, we fixed the parameters *μ* = 2^0^ and λ = 2^2^, and tested the influence of the latent variable number *k*. The latent variable numbers and AUPR scores of corresponding models are shown in Fig. 4b. Clearly, performances of SCMFDD will increase as *k* increases, and remain unchanged after reaching a threshold.

Further, we tested the impact of different similarity constraints on SCMFDD models. We have various features of drugs, and can calculate different types of drug-drug similarities, i.e. substructure similarity, target similarity, pathway similarity, enzyme similarity and drug interaction similarity. These similarities can be used as the constraint terms for SCMFDD models. We set *k* = 45%, *μ* = 2^0^ and λ = 2^2^ in the experiments. As shown in Table 2, SCMFDD models using different drug-drug similarities produce high-accuracy and robust performances. Since drug structures directly influence functions and drug interactions may induce drug effects, drug substructures and drug interactions lead to better results than other features.

**Table 2:** The performances of SCMFDD models based on different drug features

	AUPR	AUC	SN	SP	ACC	F
Substructure	0.2644	0.8737	0.3329	0.9795	0.9632	0.3130
Target	0.1947	0.8410	0.2751	0.9751	0.9575	0.2456
Pathway	0.2582	0.8706	0.3435	0.9771	0.9611	0.3079
Enzyme	0.2496	0.8671	0.3331	0.9768	0.9606	0.2990
Drug interaction	0.2638	0.8734	0.3505	0.9769	0.9611	0.3120

**Fig. 5:**
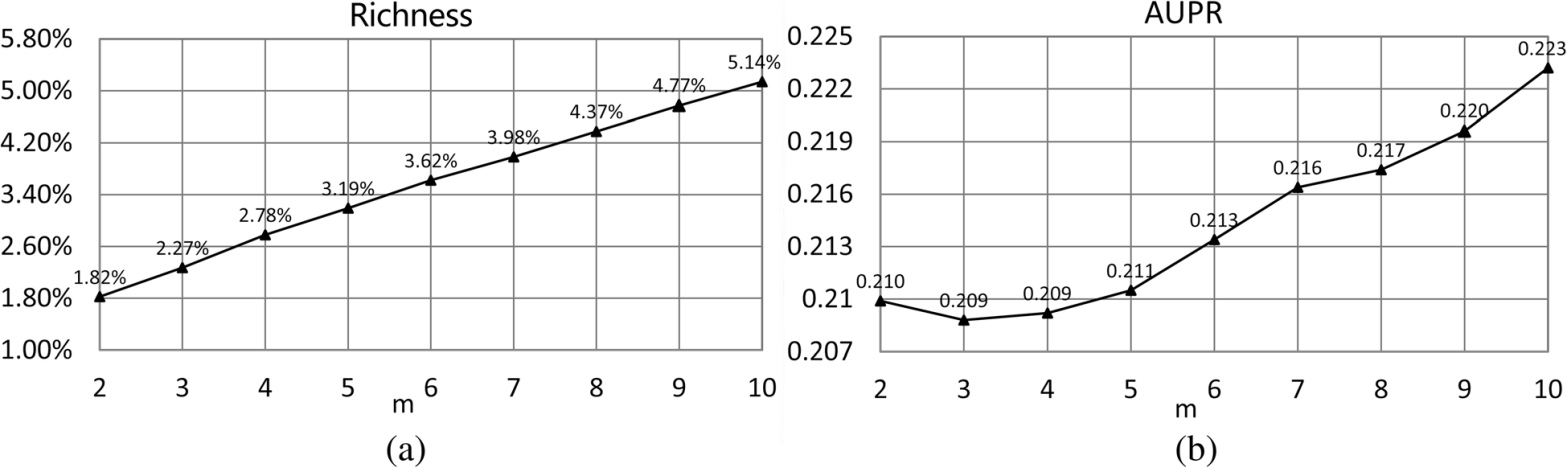
The influence of association exclusion criteria on data richness (**a**) and model performance (**b**)

The known drug-disease association is an important resource for predicting unobserved drug-disease associations. The data richness, which is the ratio of association number vs drug-disease pair number, may influence performances of SCMFDD. Here, we used the dataset SCMFDD-L for analysis. We removed drugs that are associated with less than *m* diseases, and removed diseases that associated with less than *m* drugs from SCMFDD-L dataset, *m* ∈ {2,  3, 4, 5, 6…10}. As displayed in Fig. 5, the data richness will increase as the threshold *m* increases, and then improve performances of SCMFDD models. Although the data richness influences the performances, SCMFDD could still produce robust performances.

### Comparison with state-of-the-art prediction methods

In this section, we compared our method with three state-of-the-art drug-disease association prediction methods: PREDICT [9], TL-HGBI [14] and LRSSL [18]. PREDICT constructed a universal predictor for drug repositioning to express drug-disease associations in a large-scale manner that integrates molecular structure, molecular activity and semantic data. TL-HGBI was a computational framework based on a three-layer heterogeneous network model, which made use of Omics data about diseases, drugs and drug targets to make predictions. LRSSL was a Laplacian regularized sparse subspace learning method, which integrated drug chemical information, drug target domains and target annotation information to make predictions. We obtained datasets of PREDICT [9], datasets and source codes of TL-HGBI [14] from authors. The datasets and source codes of LRSSL [18] are publicly available. Therefore, we can adopt these methods as benchmark methods for fair comparison.

First, we compared our method with PREDICT based on the PREDICT dataset by using five-fold cross validation. SCMFDD uses one drug similarity constraint and one disease similarity constraint. The PREDICT dataset contains five kinds of drug-drug similarities and two kinds of diseases-disease similarity. Thus, we built 10 different SCMFDD models by combining drug-drug similarities and diseases-disease similarities. As shown in Table 3, SCMFDD models and PREDICT produce similar AUC scores, but SCMFDD models yield much greater AUPR scores than PREDICT. Moreover, SCMFDD models were robust to different similarities, and the models based on the drug Genes-Waterman similarity and disease Gene Signature similarity produced the best results.

**Table 3:** Performance of PREDICT and SCMFDD on PREDICT Dataset

Methods	AUPR	AUC	SN	SP	ACC	F
PREDICT	0.1507	0.9020	0.3414	0.9929	0.9915	0.1437
SCMFDD-Che-GS	0.3141	0.9005	0.3663	0.9988	0.9974	0.3753
SCMFDD-Che-Phen	0.3153	0.9038	0.3678	0.9988	0.9974	0.3769
SCMFDD-SE-GS	0.3157	0.9082	0.3663	0.9988	0.9974	0.3753
SCMFDD-SE-Phen	0.3176	0.9109	0.3678	0.9988	0.9974	0.3769
SCMFDD-GP-GS	0.3210	0.9129	0.3720	0.9988	0.9975	0.3811
SCMFDD-GP-Phen	0.3224	0.9157	0.3714	0.9988	0.9975	0.3806
SCMFDD-GO-GS	0.3147	0.9035	0.3678	0.9988	0.9974	0.3769
SCMFDD-GO-Phen	0.3159	0.9065	0.3678	0.9988	0.9974	0.3769
SCMFDD-GW-GS	0.3249	0.9173	0.3389	0.9991	0.9977	0.3843
SCMFDD-GW-Phen	0.3284	0.9203	0.3776	0.9988	0.9975	0.3870

For drugs, *Che* Chemical fingerprints Similarity, *SE* Side Effect Similarity, *GP* Genes-Perlman Similarity, *GO* Genes- Ovaska Similarity, *GW* Genes-Waterman Similarity. For diseases, *GS* Gene Signature Similarity, *Phen* Phenotypic Similarity

Then, we compared our method with TL-HGBI by using TL-HGBI dataset. TL-HGBI dataset contains one drug chemical structure similarity and one disease phenotypic similarity. We constructed the SCMFDD model by using drug structure similarity and disease phenotypic similarity. As shown in Table 4, SCMFDD produced similar AUC score but much greater AUPR score compared with TL-HGBI.

Further, we compared SCMFDD and LRSSL by using LRSSL dataset. Since LRSSL dataset contains three features of drugs: chemical substructures, protein domains of target proteins, gene ontology information of target proteins. Three drug similarities were calculated, and disease semantic similarity was provided as well. Therefore, we can construct three SCMFDD models by combing three drug similarities and the disease semantic similarity. Table 5 shows the performances of prediction models evaluated by five-fold cross validation. Clearly, three SCMFDD models can produce better performance than LRSSL.

**Table 4:** Performance of TL-HGBI and SCMFDD on TL-HGBI Dataset

Methods	AUPR	AUC	SN	SP	ACC	F
TL-HGBI	0.0492	0.9584	0.1697	0.9999	0.9998	0.0840
SCMFDD	0.1500	0.9752	0.2136	0.9990	0.9990	0.0168

**Table 5:** Performance of LRSSL and SCMFDD on Liang Dataset

Methods	AUPR	AUC	SN	SP	ACC	F
LRSSL	0.1789	0.8250	0.2167	0.9989	0.9979	0.2018
SCMFDD-Che-Sem	0.2518	0.9020	0.2799	0.9993	0.9985	0.3030
SCMFDD-Dom-Sem	0.2673	0.9228	0.2851	0.9993	0.9985	0.3088
SCMFDD-Go-Sem	0.2585	0.9210	0.2897	0.9993	0.9985	0.3137

For drugs, *Che* Chemical Similarity, *Dom* Protein Domains Similarity, *Go* Gene ontology Similarity. For diseases, Sem: Semantic Similarity

### Independent experiments

In this section, we conducted independent experiments to test performances of our method in predicting novel drug-disease associations.

CTD database is an up-to-date resource about the experimentally determined drug-disease associations. Since PREDICT dataset and LRSSL dataset were compiled several years ago, we can build prediction models by using PREDICT dataset and LRSSL dataset, and check up the predictions in the CTD database. Different drugs and diseases could be matched according to their names and synonyms (provided by CTD database “Chemical vocabulary” and “Disease vocabulary”). PREDICT dataset and LRSSL dataset include different types of drug-drug similarities, and we build different similarity-based SCMFDD models for the comprehensive comparison. The PREDICT model and the LRSSL model respectively predict novel interaction by using PREDICT dataset and LRSSL dataset.

**Fig. 6:**
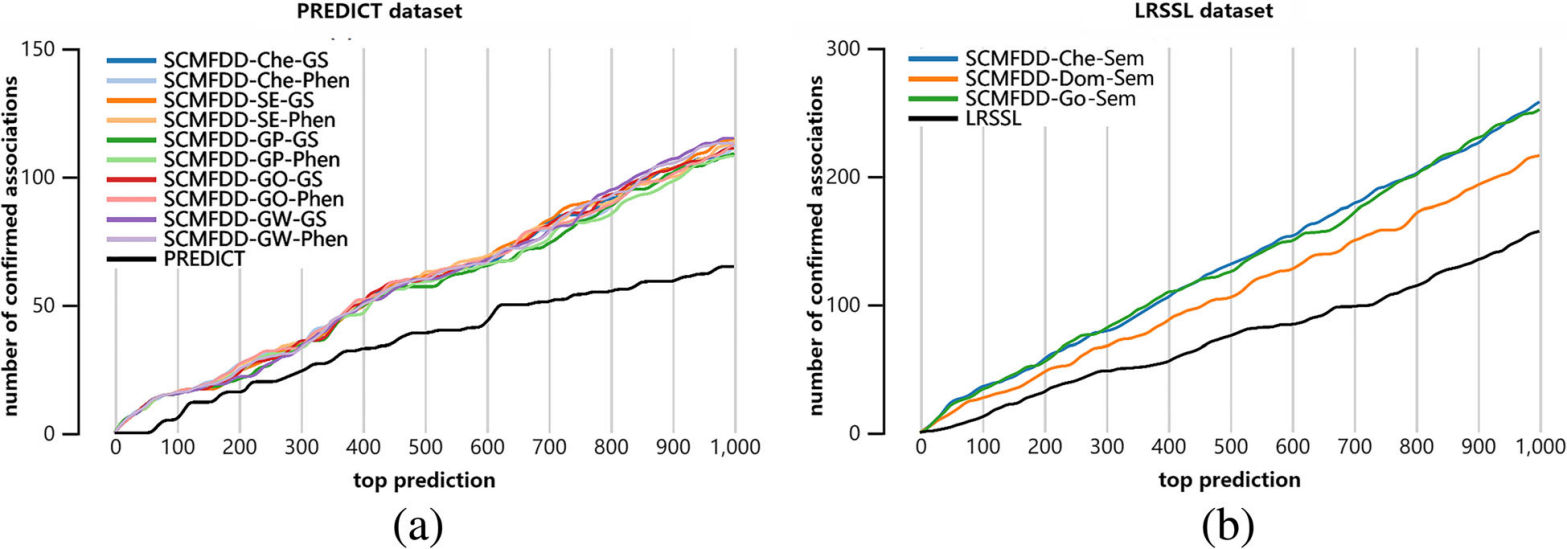
The number of confirmed associations in top predictions of PREDICT, LRSSL, SCMFDD. (a) For drugs, Che: Chemical Similarity, SE: Chemical Similarity, GP: Genes-Perlman Similarity, GO: Genes- Ovaska Similarity, GW: Genes-Waterman Similarity. For diseases, GS: Gene Signature Similarity, Phen: Phenotypic Similarity (b) For drugs, Che: Chemical Similarity, Dom: Protein Domains Similarity, Go: Gene ontology Similarity. For diseases, Sem: Semantic Similarity

We considered the top predictions from top 2 to top 1000 in a step size of 2, and respectively counted how many predicted associations can be confirmed in CTD database. Figure 6 shows the number of checked predictions and the number of confirmed associations. Clearly, our method finds out more novel associations than benchmark methods, and has the good performances in the independent experiments.

### Web server and applications

To facilitate the drug-disease association prediction, we developed a web server named “SCMFDD” by using the dataset SCMFDD-L, available at http://www.bioinfotech.cn/SCMFDD/. Users can predict novel drug-disease associations for a given drug or a given disease, and then visualize predictions. Here, we used two case studies to illustrate the usefulness for the drug-disease association prediction of our web server.

Clozapine is an effective drug to treat patients with refractory schizophrenia [39, 40]. Clozapine works by changing the actions of chemicals in the brain. Here, the web server predicts diseases that are associated with Clozapine. Table 6 lists top 10 predictions among all unknown relationships between Clozapine and diseases in the SCMFDD-L dataset. Then, we analyze these predicted diseases case by case. From https://en.wikipedia.org/wiki/Clozapine (access on 2018–2-1), three diseases: sleep initiation and maintenance disorders (also insomnia), status epilepticus and headache have been reported as side effects of Clozapine, indicating that they have associations with the drug “Clozapine”. Further, the study [41] found that Clozapine improved the syndrome of inappropriate antidiuretic hormone secretion(SIADH) in a patient; the studies [42, 43] revealed that Clozapine can be used for the treatment of post-traumatic stress disorder (PTSD); the study [44] demonstrated that Clozapine can be used for the treatment of Parkinson’s disease; the study [45] indicated that Clozapine can affect the visual memory.

**Table 6:** Top 10 predicted diseases associated with Clozapine

Index	Disease Name	Disease ID	Score	Evidence
1	Sleep Initiation and Maintenance Disorders	D007319	1	https://en.wikipedia.org/wiki/Clozapine
2	Anxiety Disorders	D001008	0.9117	N.A.
3	Inappropriate ADH Syndrome	D007177	0.7434	A Case report [41]
4	Stress Disorders, Post-Traumatic	D013313	0.7267	Report [42, 43]
5	Parkinson Disease, Secondary	D010302	0.7179	Review [44]
6	Memory Disorders	D008569	0.7123	An animal study [45]
7	Status Epilepticus	D013226	0.6312	https://en.wikipedia.org/wiki/Clozapine
8	Headache	D006261	0.6166	https://en.wikipedia.org/wiki/Clozapine
9	Torsades de Pointes	D016171	0.5953	N.A.
10	Attention Deficit Disorder with Hyperactivity	D001289	0.5913	N.A.

Scores are normalized by using ((score-min)/(max-min))

Alzheimer’s disease (AD) is a chronic neurodegenerative disorder that leads to disturbances of cognitive functions. The radical cause and effective treatment of AD remain unclear, and AD has attracted many scientists to study its pathogenic mechanism and therapeutic function. Table 7 lists top 10 predicted drugs associated with Alzheimer’s disease, and evidence is available for six drugs. For example, the study [46] revealed that Olanzapine appears to be effective in treating psychotic and behavioral disturbances associated with AD; the study [47] found that stimulation of the dopaminergic system could improve cognitive function in a murine model and suggested that Levodopa that works in the dopaminergic system could ameliorate typical symptoms of AD: learning and memory deficits. The study [48] revealed that the presence of Malondialdehyde level is a risk factor for AD. The study [49] confirmed that progesterone significantly could reduce and inhibit tau hyperphosphorylation, a chemical process implicated in AD. The study [50] demonstrated that Valproic Acid (VPA) could decrease β-amyloid(Aβ) production which is the key risk factor in AD and improve memory deficits of AD model mice. The study [51] showed that Ethanol protect neurons against Aβ-induced synapse damage and explained epidemiological reports that moderate alcohol consumption protects against the development of AD.

**Table 7:** Top 10 predicted drugs associated with Alzheimer’s disease

Index	Drug Name	Drug MeSH ID	DrugBank ID	PubChem CID	Score(normalized)	Evidence
1	Nitroprusside	D009599	DB00325	11,963,622	1	N.A.
2	Tamoxifen	D013629	DB00675	2,733,526	0.7644	N.A.
3	Olanzapine	C076029	DB00334	4585	0.7269	A clinical study [46]
4	Sucralfate	D013392	DB00364	70,789,197	0.7223	N.A.
5	Levodopa	D007980	DB01235	6047	0.6893	An animal study [47]
6	Malondialdehyde	D008315	DB03057	10,964	0.6767	A clinical study [48]
7	Progesterone	D011374	DB00396	5994	0.6695	An animal study [49]
8	Valproic Acid	D014635	DB00313	3121	0.6625	An animal study [50]
9	Scopolamine Hydrobromide	D012601	DB00747	3,000,322	0.6522	N.A.
10	Ethanol	D000431	DB00898	702	0.6402	A clinical study [51]

Scores are normalized by using ((score-min)/(max-min))

**Fig. 7:**
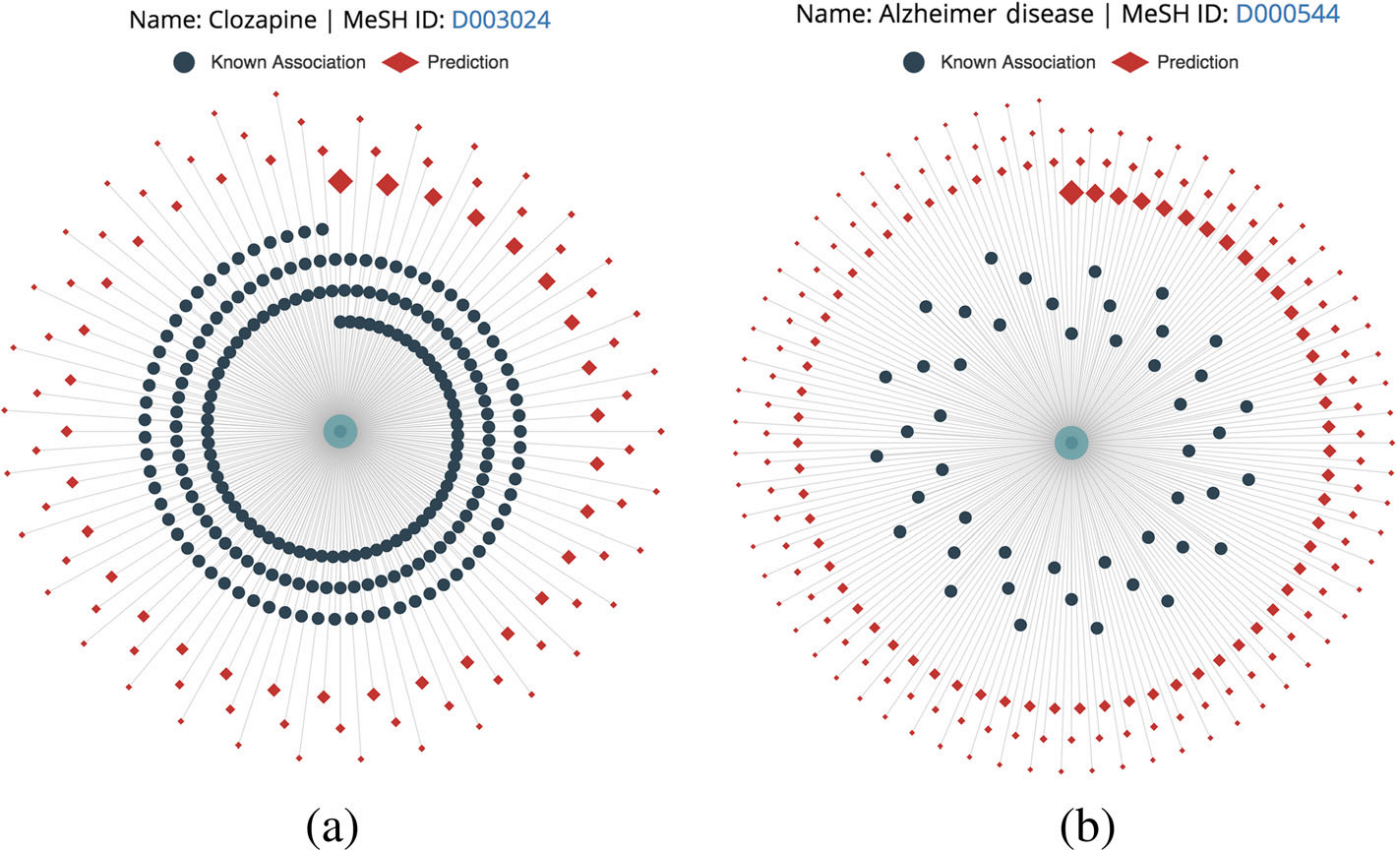
Web Visualization of predictions for Clozapine **a** and predictions for Headache **b**

The server can visualize the predictions. Figure 7 shows the top 100 predictions for Clozapine and top 200 predictions for Alzheimer’s disease. As shown in Fig. 7a, “dark blue circle” stands for a disease, which has a known association with Clozapine, and “red square” stands for predicted diseases, which have an association with Clozapine. As shown in Fig. 7b, “dark blue circle” stands for a drug, which has a known association with Alzheimer’s disease, and “red square” stands for predicted drugs, which have an association with Alzheimer’s disease. Users can adjust the number of predictions for visualization.

## Conclusion

In this paper, we proposed a computational method “SCMFDD” to predict unobserved drug-disease associations. SCMFDD incorporate drug feature-based similarities and disease semantic similarity into the matrix factorization frame. Experimental results show that SCMFDD can produce high-accuracy performances on five benchmark datasets when evaluated by five-fold cross validation, and SCMFDD outperforms state-of-the-art methods under fair comparison. Moreover, SCMFDD produces satisfying performances for different similarity constraints, and is also robust to the data richness. We constructed a web server based on drug-disease associations, which are collected from the CTD database. The server can predict novel drug-disease associations, and also can help researchers to quickly find associations for interested drugs or diseases.

In recent years, the deep learning methods have been applied to similar tasks [52–54]. However, designing an effective neural network is a hard task, and the training process also costs a great amount of time. Compared to deep learning-based methods, SCMFDD is easy to implement, and SCMFDD can be applied into similar tasks in bioinformatics.

However, SCMFDD still has several limitations. First, SCMFDD has three parameters, and there is no good way of determining suitable parameters except going through all combinations. For our datasets, it costs dozens of hours to determine optimal parameters. Second, SCMFDD only uses individual drug feature-based similarity to build prediction models. When we have multiple drug features, we can calculate different drug feature-based similarities. Combining diverse information can usually lead to improved performances [55–60], and how to integrate multiple similarities in a model is our future work. Third, the server can make predictions for the drugs and diseases in our dataset, but can’t support other drugs or diseases.

## Abbreviations

AUC: Area under ROC curve
AUPR: Area under the precision-recall curve
SCMFDD: Similarity constrained matrix factorization method

## References

[1] Wilson JF (2006). Alterations in processes and priorities needed for new drug development. Ann Intern Med.

[2] Dimasi JA (2001). New drug development in the United States from 1963 to 1999. Clin Pharmacol Ther.

[3] Adams CP, Brantner VV (2006). Estimating the cost of new drug development: is it really 802 million dollars?. Health Aff.

[4] Davis AP, Grondin CJ, Johnson RJ, Sciaky D, King BL, McMorran R, Wiegers J, Wiegers TC, Mattingly CJ (2017). The comparative Toxicogenomics database: update 2017. Nucleic Acids Res.

[5] von Eichborn J, Murgueitio MS, Dunkel M, Koerner S, Bourne PE, Preissner R (2011). PROMISCUOUS: a database for network-based drug-repositioning. Nucleic Acids Res.

[6] Wang L, Wang Y, Hu Q, Li S (2014). Systematic analysis of new drug indications by drug-gene-disease coherent subnetworks. CPT: pharmacometrics & systems pharmacology.

[7] Wiegers TC, Davis AP, Cohen KB, Hirschman L, Mattingly CJ (2009). Text mining and manual curation of chemical-gene-disease networks for the comparative toxicogenomics database (CTD). BMC Bioinformatics.

[8] Yu L, Huang J, Ma Z, Zhang J, Zou Y, Gao L. Inferring drug-disease associations based on known protein complexes. BMC Med Genet. 2015;8(Suppl 2, S2) 10.1186/1755-8794-8-S2-S2 PMC4460611 26044949

[9] Gottlieb A, Stein GY, Ruppin E, Sharan R (2011). PREDICT: a method for inferring novel drug indications with application to personalized medicine. Mol Syst Biol.

[10] Yang L, Agarwal P (2011). Systematic drug repositioning based on clinical side-effects. PLoS One.

[11] Wang Y, Chen S, Deng N, Wang Y (2013). Drug repositioning by kernel-based integration of molecular structure, molecular activity, and phenotype data. PLoS One.

[12] Huang YF, Yeh HY, Soo VW (2013). Inferring drug-disease associations from integration of chemical, genomic and phenotype data using network propagation. BMC Med Genet.

[13] Oh M, Ahn J, Yoon Y (2014). A network-based classification model for deriving novel drug-disease associations and assessing their molecular actions. PLoS One.

[14] Wang W, Yang S, Zhang X, Li J (2014). Drug repositioning by integrating target information through a heterogeneous network model. Bioinformatics.

[15] Martinez V, Navarro C, Cano C, Fajardo W, Blanco A (2015). DrugNet: network-based drug-disease prioritization by integrating heterogeneous data. Artif Intell Med.

[16] Wang H, Gu Q, Wei J, Cao Z, Liu Q (2015). Mining drug-disease relationships as a complement to medical genetics-based drug repositioning: where a recommendation system meets genome-wide association studies. Clin Pharmacol Ther.

[17] Moghadam H, Rahgozar M, Gharaghani S (2016). Scoring multiple features to predict drug disease associations using information fusion and aggregation. SAR QSAR Environ Res.

[18] Liang X, Zhang P, Yan L, Fu Y, Peng F, Qu L, Shao M, Chen Y, Chen Z (2017). LRSSL: predict and interpret drug–disease associations based on data integration using sparse subspace learning. Bioinformatics.

[19] Li Q, Cheng T, Wang Y, Bryant SH (2010). PubChem as a public resource for drug discovery. Drug Discov Today.

[20] Law V, Knox C, Djoumbou Y, Jewison T, Guo AC, Liu Y, Maciejewski A, Arndt D, Wilson M, Neveu V (2014). DrugBank 4.0: shedding new light on drug metabolism. Nucleic Acids Res.

[21] Kanehisa M, Goto S, Furumichi M, Tanabe M, Hirakawa M (2010). KEGG for representation and analysis of molecular networks involving diseases and drugs. Nucleic Acids Res.

[22] Smith TF, Waterman MS, Burks C (1985). The statistical distribution of nucleic acid similarities. Nucleic Acids Res.

[23] Perlman L, Gottlieb A, Atias N, Ruppin E, Sharan R (2011). Combining drug and gene similarity measures for drug-target elucidation. J Comput Biol.

[24] Ovaska K, Laakso M, Hautaniemi S (2008). Fast gene ontology based clustering for microarray experiments. BioData Min.

[25] Wang F, Zhang P, Cao N, Hu J, Sorrentino R (2014). Exploring the associations between drug side-effects and therapeutic indications. J Biomed Inform.

[26] Xuan P, Han K, Guo M, Guo Y, Li J, Ding J, Liu Y, Dai Q, Li J, Teng Z (2013). Prediction of microRNAs associated with human diseases based on weighted k most similar neighbors. PLoS One.

[27] Chen X, Yan CC, Luo C, Ji W, Zhang Y, Dai Q (2015). Constructing lncRNA functional similarity network based on lncRNA-disease associations and disease semantic similarity. Sci Rep.

[28] Wang D, Wang J, Lu M, Song F, Cui Q (2010). Inferring the human microRNA functional similarity and functional network based on microRNA-associated diseases. Bioinformatics.

[29] Ma Y, Fu Y (2012). Manifold learning theory and applications.

[30] Zhang W, Liu X, Chen Y, Wu W, Wang W, Li X (2018). Feature-derived graph regularized matrix factorization for predicting drug side effects. Neurocomputing.

[31] Rana B, Juneja A, Saxena M, Gudwani S, Kumaran SS, Behari M, Agrawal RK (2015). Graph-theory-based spectral feature selection for computer aided diagnosis of Parkinson's disease using T1-weighted MRI International Journal of Imaging Systems and Technology Volume 25, Issue 3. Int J Imaging Syst Technol.

[32] Chung FRK: Spectral graph theory. Providence, R.I.: published for the conference board of the mathematical sciences by the American Mathematical Society; 1997.

[33] Zhang W, Chen Y, Li D (2017). Drug-target interaction prediction through label propagation with linear neighborhood information. Molecules.

[34] Zhang W, Qu Q, Zhang Y, Wang W (2018). The linear neighborhood propagation method for predicting long non-coding RNA–protein interactions. Neurocomputing.

[35] Zhang W, Yue X, Chen Y, Lin W, Li B, Liu F, Li X. Predicting drug-disease associations based on the known association bipartite network. IEEE Int Conf Bioinformatics Biomed. 2017:503–9.

[36] Zhang W, Chen Y, Tu S, Liu F, Qu Q. Drug side effect prediction through linear neighborhoods and multiple data source integration. IEEE Int C Bioinform. 2016:427–34.

[37] Ruan CY, Wang Y, Zhang YC, Ma JG, Chen HJ, Aickelin U, Zhu SF, Zhang T (2017). THCluster:herb supplements categorization for precision traditional Chinese medicine. IEEE Int Conf Bioinformatics And Biomedicine.

[38] Zhang W, Yue X, Liu F, Chen YL, Tu SK, Zhang XN. A unified frame of predicting side effects of drugs by using linear neighborhood similarity. BMC Syst Biol. 2017;11 10.1186/s12918-017-0477-2 PMC5751767 29297371

[39] Alvir JM, Lieberman JA, Safferman AZ, Schwimmer JL, Schaaf JA (1993). Clozapine-induced agranulocytosis. Incidence and risk factors in the United States. N Engl J Med.

[40] Lieberman JA, Alvir JM (1992). A report of clozapine-induced agranulocytosis in the United States. Incidence and risk factors. Drug Saf.

[41] Fujimoto M, Hashimoto R, Yamamori H, Yasuda Y, Ohi K, Iwatani H, Isaka Y, Takeda M (2016). Clozapine improved the syndrome of inappropriate antidiuretic hormone secretion in a patient with treatment-resistant schizophrenia. Psychiatry Clin Neurosci.

[42] Abejuela HR, Festin FE, Lynn E. Clozapine for Treatment- Resistant Post-Traumatic Stress Disorder (PTSD). J Traum Stress Disord Treatment. 2014;3(2):1–9.

[43] Kant R, Chalansani R, Chengappa KN, Dieringer MF (2004). The off-label use of clozapine in adolescents with bipolar disorder, intermittent explosive disorder, or posttraumatic stress disorder. J Child Adolesc Psychopharmacol.

[44] Klein C, Gordon J, Pollak L, Rabey JM (2003). Clozapine in Parkinson's disease psychosis: 5-year follow-up review. Clin Neuropharmacol.

[45] Mutlu O, Ulak G, Celikyurt IK, Akar FY, Erden F, Tanyeri P (2011). Effects of olanzapine, sertindole and clozapine on MK-801 induced visual memory deficits in mice. Pharmacol Biochem Behav.

[46] Schatz RA (2003). Olanzapine for psychotic and behavioral disturbances in Alzheimer disease. Ann Pharmacother.

[47] Ambrée O, Richter H, Sachser N, Lewejohann L, Dere E, Ma DSS, Herring A, Keyvani K, Paulus W, Schäbitz WR (2009). Levodopa ameliorates learning and memory deficits in a murine model of Alzheimer's disease. Neurobiol Aging.

[48] Lópezriquelme N, Alompoveda J, Vicianomorote N, Llinaresibor I, Tormodíaz C (2016). Apolipoprotein E ε4 allele and malondialdehyde level are independent risk factors for Alzheimer’s disease. SAGE Open Med.

[49] Carroll JC, Rosario ER, Chang L, Stanczyk FZ, Oddo S, Laferla FM, Pike CJ (2007). Progesterone and estrogen regulate Alzheimer-like neuropathology in female 3xTg-AD mice. J. Neurosci. Off. J. Soc. Neurosci.

[50] Hong Q, He G, Ly PTT, Fox CJ, Staufenbiel M, Cai F, Zhang Z, Wei S, Sun X, Chen CH (2008). Valproic acid inhibits Aβ production, neuritic plaque formation, and behavioral deficits in Alzheimer's disease mouse models. J Exp Med.

[51] Bate C, Williams A (2011). Ethanol protects cultured neurons against amyloid-β and α-synuclein-induced synapse damage. Neuropharmacology.

[52] Cohen T, Widdows D (2017). Embedding of semantic predications. J Biomed Inform.

[53] Mower J, Subramanian D, Shang N, Cohen T (2016). Classification-by-analogy: using vector representations of implicit relationships to identify plausibly causal drug/side-effect relationships. AMIA Annu Symp Proc.

[54] Zhang W, Zhu X, Fu Y, Tsuji J, Weng Z (2017). Predicting human splicing branchpoints by combining sequence-derived features and multi-label learning methods. BMC Bioinformatics.

[55] Zhang W, Niu Y, Zou H, Luo L, Liu Q, Wu W (2015). Accurate prediction of immunogenic T-cell epitopes from epitope sequences using the genetic algorithm-based ensemble learning. PLoS One.

[56] Zhang W, Liu F, Luo L, Zhang J (2015). Predicting drug side effects by multi-label learning and ensemble learning. BMC Bioinformatics.

[57] Li D, Luo L, Zhang W, Liu F, Luo F (2016). A genetic algorithm-based weighted ensemble method for predicting transposon-derived piRNAs. BMC Bioinformatics.

[58] Luo L, Li D, Zhang W, Tu S, Zhu X, Tian G. Accurate prediction of transposon-derived piRNAs by integrating various sequential and physicochemical features. PLoS One. 2016;11(4). 10.1371/journal.pone.0153268 PMC4830532 27074043

[59] Zhang W, Chen YL, Liu F, Luo F, Tian G, Li XH. Predicting potential drug-drug interactions by integrating chemical, biological, phenotypic and network data. Bmc Bioinformatics. 2017;18:18. 10.1186/s12859-016-1415-9 PMC5217341 28056782

[60] Zhang W, Shi JW, Tang GF, Wu WJ, Yue X, Li DF (2017). Predicting small RNAs in bacteria via sequence learning ensemble method. IEEE Int Conf Bioinformatics Biomed.

